# Tuning Ni–MoO_2_ Catalyst–Ionomer and Electrolyte Interaction for Water Electrolyzers with Anion Exchange Membranes

**DOI:** 10.1021/acsaem.0c03072

**Published:** 2021-03-23

**Authors:** Alaa Y. Faid, Alejandro Oyarce Barnett, Frode Seland, Svein Sunde

**Affiliations:** † Department of Materials Science and Engineering, Norwegian University of Science and Technology, 7491, Trondheim, Norway; ‡ SINTEF Industry, New Energy Solutions Department, 7465, Trondheim, Norway; § Department of Energy and Process Engineering, Norwegian University of Science and Technology, 7491, Trondheim, Norway

**Keywords:** catalyst-ionomer interaction, catalyst-electrolyte interaction, anion exchange ionomer, anion exchange membrane, hydrogen evolution, nickel catalysts, water electrolysis

## Abstract

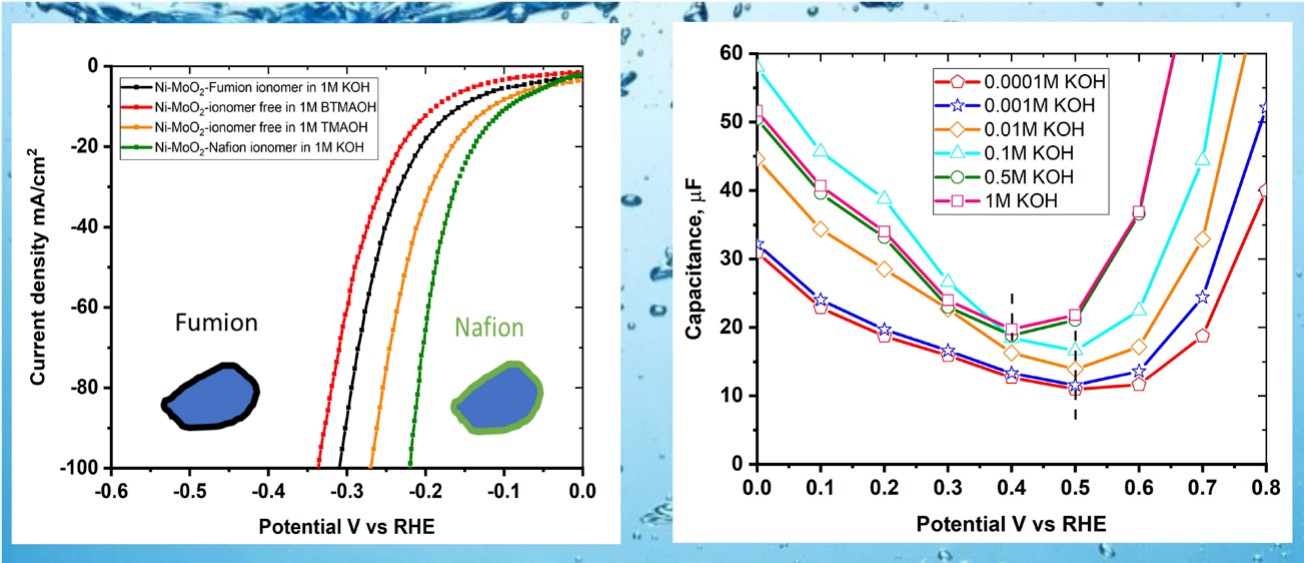

Tailoring catalyst–ionomer and electrolyte interaction is crucial for the development of anion exchange membrane (AEM) water electrolysis. In this work, the interaction of Ni–MoO_2_ nanosheets with ionomers and electrolyte cations was investigated. The activity of Ni–MoO_2_ nanosheets for the hydrogen evolution reaction (HER) increased when tested in 1 M NaOH compared to 1 M KOH; however, it decreased when tested in 0.01 M KOH compared to 1 M KOH electrolyte. The capacitance minimum associated with the potential of zero free charge (pzfc) was shifted negatively from 0.5 to 0.4 V versus RHE when KOH concentration increased from 0.1 mM to 1 M KOH, suggesting a softening of the water in the double-layer to facilitate the OH^–^ transport and faster kinetics of the Volmer step that lead to improved HER activity. The catalyst interaction with cationic moieties in the anion ionomer (or organic electrolytes) can also be rationalized based on the capacitance minimum, because the latter indicates a negatively charged catalyst during the HER, attracting the cationic moieties leading to the blocking of the catalytic sites and lower HER performance. The HER activity of Ni–MoO_2_ nanosheets is lower in benzyltrimethylammonium hydroxide (BTMAOH) than in tetramethylammonium hydroxide (TMAOH). Anion fumion ionomer and electrolytes with organic cations with benzyl group adsorption (such as BTMAOH) lead to decreased HER activity in comparison with TMAOH and Nafion. By utilizing Ni–MoO_2_ nanosheet electrodes as a cathode in a full non-platinum group metal (PGM) AEM electrolyzer, a current density of 1.15 A/cm^2^ at 2 V cell voltage in 1 M KOH at 50 °C was achieved. The electrolyzer showed exceptional stability in 0.1 M KOH for 65 h at 0.5 A/cm^2^.

## Introduction

Hydrogen is a chemical feedstock for chemical synthesis and fuel for transportation and energy storage.^1^ Using renewable energy for water electrolysis represents a competitive approach for sustainable generation of hydrogen.^2,3^ Anion exchange membrane (AEM) electrolyzers aim to bring the merits of proton exchange membrane (PEM) electrolysis (operating at differential pressure, dynamic response, and higher current densities) and liquid alkaline systems (low cost and stability).^3−5^ AEM electrolyzer systems can use cheaper catalysts/electrodes and a balance of plant components while also having the potential for achieving high efficiency.^6^

The hydrogen evolution reaction (HER) kinetics is sluggish in an alkaline environment with 2 orders of magnitude slower than in an acidic environment for Pt-based catalysts.^7^ The large activation energy (*E*_a_) of the H_ads_ intermediate formation (Volmer reaction) has been suggested to be the cause for the slower HER kinetics in alkaline electrolytes.^8,9^

The HER catalyst–ionomer–electrolyte interaction in AEM electrolysis is crucial for the development of electrolyzer devices.^8^ The literature reports that anion exchange ionomers lead to lower HER performance in comparison with Nafion ionomer.^4,8^ The difference in performance has been associated with the effects of ionomer cationic groups and ionomer polymer backbone chemistry (poly(arylene ethers), polybenzimidazole (PBI), etc.).^5,10^ Also, the electrolyte cation influences the HER activity, for example, Pt HER activity increases by a factor of 4 going from Cs^+^ through Rb^+^, K^+^, Na^+^, to Li^+^.^11^ Finally, the electrolyte concentration appears to influence the HER activity of nanostructured catalysts with the performance of HER increasing with increasing the concentration of KOH.^12,13^ Wang et al. have shown that the improved HER performance with increasing the concentration of KOH is related to in situ H_3_O^+^ intermediates that generated on the surface of nanocatalyst.^14^

The influence of the ionomer and electrolyte on catalyst performance may be broadly classified as due to either covalent interaction or electrostatic effects.^8,15^ Depending on the charge carried by the electrode itself, the charged species in the ionomer and electrolyte may, therefore, be repelled or attracted to the electrode electrostatically and in the latter case thus affect the HER activity. The charge on the electrode, in turn, may be assessed from the difference between the electrode potential and its potential of zero charge (pzc). The pzc is associated with the potential at which there is no charge stored at the electrode–electrolyte interface.^16,17^ The pzc can be specified as representing the free charge or the total charge. The potential of zero free charge (pzfc) is the potential at which the metal surface at its interface to the electrolyte has zero free (electron) charge. The potential of zero total charge (pztc) is the potential at which the electrode total charge, including any charge associated with adsorbed surface species, equals zero. The pztc depends on the electrode material, adsorbed species, and electrolyte.^18,19^

According to the Stern model, the merger of Gouy–Chapman (GC) theory and the Helmholz models of double layer, the capacitance (*C*) will be minimum at the pzc.^16,17,20,21^ In this work, we will assume that changes in the electrode capacitance as measured by impedance spectroscopy reflect changes in the pzfc. For a pure electrostatic interaction between the surface of the electrode and charged species, when *E* > *E*_pzc_ anions will be attracted, whereas cations and quaternary ammonium moieties in state-of-the-art AEM ionomers will be adsorbed or at least attracted to the electrode when *E* < *E*_pzc_.^19^ The pzc is a fundamental characteristic of the catalyst–electrolyte interface, critical for a detailed understanding of the double layer phenomenon.^19^

The so-called pzfc theory^22,23^ relates the activity for the HER to rates of the OH^–^ transport through the double-layer at the electrode–electrolyte interface. Ledezma-Yanez et al. found that a positive shift in the pzfc induces large reorganization energy of the interfacial water that leads to more structured water networks and hinders both the transfer of OH^–^ at the interface and the Volmer step kinetics.^23^ By depositing Ni hydroxide on Pt (111) surface, this causes shifting of pzfc closer to the hydrogen adsorption potential, which indicates a reduction in the hydrogen adsorption activation barrier and boosting of the HER performance.^22^

NiMo alloys and compounds have shown great potential as a cathode catalyst in alkaline electrolysis.^24^ A Ni content of 60–80% in these alloys, in addition to different metal oxides such as MoO_2_ and MoO_3_, results in superior HER activity due to modified d-band electron states, availability of exposed active sites, and rapid electron transfer that accelerate the rates of hydrogen adsorption and desorption.^25−28^

In this work, an active HER catalyst (Ni–MoO_2_ nanosheets) was used to evaluate catalyst–electrolyte–ionomer interaction for AEM water electrolysis. The influence of KOH concentration, inorganic electrolyte cations, the type of ionomer, and the presence of organic electrolytes with cations were studied and analyzed. The capacitance minimum, related to the pzfc, has been measured in various KOH concentrations (0.1 mM to 1 M KOH) and used to rationalize the activity with respect to the HER in terms of catalyst–electrolyte–ionomer interaction. Finally, we describe the performance and stability of a full non-PGM AEM electrolyzer with Ni–MoO_2_ nanosheet catalyst as the cathode.

## Experimental Section

### Catalyst Synthesis

For Ni–MoO_2_ nanosheet synthesis, the NiMo precursors are chemically reduced by sodium borohydride where 25 mM of NiMo precursors [nickel nitrate hexahydrate Ni(NO_3_)_2_·6H_2_O (crystallized, ≥97.0%, Sigma-Aldrich) and sodium molybdate, (≥98%, Sigma-Aldrich)] (20 wt % molybdenum in precursors) were mixed in 0.5 L water (resistivity = 18.2 MΩ cm, Milli-Q ultrapure water). One liter of 75 mM sodium borohydride (NaBH_4_) was added once to the precursors’ solution and the solution turned black. To ensure the precursor’s complete reduction, the solution mixture was stirred for 60 min. The black precipitate was collected by centrifugation five times for 6 min at 8000 rpm, cleaned with ethanol and water mixture (ethanol was used to remove any synthesis contamination and leftover precursors), and then dried in a vacuum oven overnight at 60 °C. Subsequent thermal treatment resulted in an oxide, alloy, or mixture of both. The catalyst powder of Ni with 20% molybdenum was annealed for 2 h in 5% H_2_/Ar atmosphere at 500 °C with a temperature increase rate of 10 °C/min to get Ni–MoO_2_ nanosheets. Ni nanosheet catalyst was prepared in the same way as above using nickel nitrate precursor only.

### Structural and Electrochemical Characterization

Catalyst morphology was studied using (Carl Zeiss supra 55) scanning electron microscopy (SEM) while catalyst composition analysis was carried out using energy dispersive X-ray (EDX) spectroscopy. The morphology of catalysts was further analyzed by scanning transmission electron microscopy (STEM) in a Hitachi S-5500 device. The catalyst crystallinity and phases were examined using X-ray diffraction (XRD) using Bruker device (Cu–Kα, λ = 1.5425 Å). X-ray photoelectron spectroscopy (XPS) was used as a tool to explore catalyst surface composition using Kratos Axis Ultra DLD device (monochromatic Al X-ray).

The catalysts electrochemical investigation was conducted in a three-electrode cell where the Hg/HgO electrode and graphite rod served as the reference and counter electrode, respectively. The working electrode was a 5 mm diameter glassy carbon (GC) electrode. All electrodes were purchased from Pine Research. The working electrode was rotated at 1600 rpm using a rotator purchased from PINE Research. The electrochemical data were collected using a potentiostat (Ivium-n-Stat). The polishing procedure of the GC electrode was done using both 5 and 0.05 μm Al_2_O_3_ suspension. After polishing, the electrode was thoroughly washed with water, sonicated for 5 min in 1 M KOH, and then rinsed thoroughly again with water. The inks from catalyst powders were made by adding 10 mg of catalyst powder in a solution of 1 mL of ethanol and 50 μL of Nafion ionomer (Sigma-Aldrich, 5 wt %) or 25 μL of Fumion FAA-3 ionomer (10 wt %, fuel cell store). 60% Pt/C (Alfa Aesar) was used to compare catalyst interaction with Ni-MoO_2_ nanosheets. The ink was sonicated in an ice bath for 30 min before being deposited on the GC electrode with mass loading of 0.2 mg/cm^2^ (based on total catalyst mass) unless otherwise stated. The alkaline electrolytes used in this work were N_2_-saturated at room temperature (20 ± 2 °C). To compare the effect of the electrolyte cation, 1 M KOH or NaOH (Sigma-Aldrich) was used. The HER activity was evaluated in various concentrations (1, 0.1, and 0.01 M) of KOH (85%, Sigma-Aldrich), and the procedure reported by Trotochaud et al.^29^ was used when purification was needed.

The working electrode was activated until getting reproducible cyclic voltammograms (CVs) after 50 cycles in a potential range of −0.8 to −1.3 V versus Hg/HgO at a 100 mV/s scan rate. The linear sweep voltammograms (LSV) was collected in the same potential range as CVs but at a sweep rate of 1 mV/s. We used a similar approach as in literature^12,30^ to calibrate the Hg/HgO electrode to reversible hydrogen electrode (RHE). The calibration approach was carried out in a hydrogen-saturated electrolyte using Pt electrodes as working and counter electrodes while using our Hg/HgO as the reference electrode. By collecting HER LSV curves at 1 mV/s and measuring the potential at zero current, the Hg/HgO can be calibrated against RHE. The Hg/HgO reference electrode in 1 M KOH was calibrated to RHE using the experimentally measured value as follows
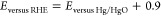
1Electrochemical impedance spectroscopy (EIS) was collected in a 10^5^– 0.1 Hz frequency range with 10 mV (rms) perturbation amplitude at specific overpotentials. The compensation of electrode potential was carried out using cell ohmic drop (*R*) obtained from EIS at high frequency as in the following equation
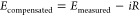
2where *E*_measured_ and *E*_compensated_ are the measured and compensated potentials in V, respectively, while *i* is the current in A, and *R* is the resistance in Ω.

Chronoamperometry responses were collected for 30 h at −0.35 V versus RHE. An accelerated stress test (AST) was used to further assess catalyst stability and durability. The AST procedure includes electrode cycling for 5000 cycles in a potential range from 0.1 to −0.4 V versus RHE at a 100 mV/s scan rate.

### Effect of Quaternary Ammonium

The HER activity was investigated in various N_2_-saturated electrolytes such as benzyl trimethylammonium hydroxide solution (BTMAOH), tetramethylammonium hydroxide (TMAOH), and a combination of BTMAOH and KOH. The pH of the combined (KOH + BTMAOH) electrolyte was kept constant at 13.95 by using *Y* M KOH + (1 – *Y*) M BTMAOH. For the ionomer-free catalyst layer RDE, the catalyst (Ni–MoO_2_ nanosheets and 60% Pt/C) was suspended in a solution mixture of water and isopropanol in an ultrasonic bath. The ink was deposited on the GC electrode and dried under an ambient environment. The working electrode was activated for 50 cycles in a potential range from 0.1 to −0.4 V versus RHE at a 100 mV/s scan rate before collecting the LSV polarization curves at 1 mV/s sweep rate in the 0.1 to −0.4 V versus RHE potential range.

### Potential of Zero Charge

The capacitance was measured using electrochemical impedance spectroscopy at different potentials in the range from −0.1 to 1.4 V versus RHE applying frequency from 10 Hz to 1 kHz with 5 mV perturbation amplitude. A similar method has been used previously in the literature.^31,21,32^ The measurements were carried out in various KOH concentrations from 0.1 mM to 1 M KOH.

### Membrane Electrode Assembly (MEA)

**Figure 1:**
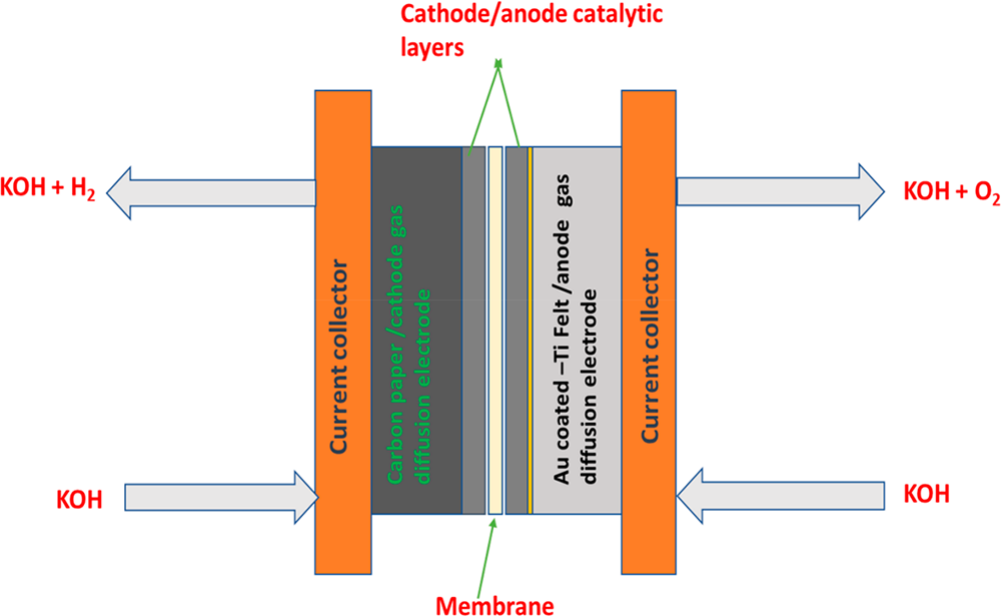
Schematic representation of the AEM water electrolyzer setup including the membrane electrode assembly used in this work. Figure reproduced with permission from ref (12). Copyright 2021, Elsevier.

The catalyst inks for cathode were prepared by dispersing (Ni–MoO_2_ nanosheets) catalyst powder in a solution mixture of water/isopropanol (1:1) solvents and ionomer. The ink solution was then sonicated in an ice bath for 30 min. Anode catalyst ink was made of a laboratory optimized NiCoFe (Ni_0.6_Co_0.2_Fe_0.2_) catalyst. Catalyst layers were fabricated as catalyst-coated substrates (CCSs) by spraying cathode inks on a 25 cm^2^ carbon paper (Toray 090, fuel cell store), whereas anode inks were sprayed on a 25 cm^2^ Au coated-Ti felt (Bekaert) using Coltech airbrush (1 bar pressure) at 60 °C. To clean Ti felt and reduce cell contact resistance, the Ti felt was pretreated to eliminate surface oxide by etching for 2 min in HCl (37 wt %, Sigma-Aldrich) and sonicated in a mixture of water and ethanol for 5 min before being introduced to Edwards sputtering machine to be coated with Au. The Au coating was done for 2 min on each side at 20 mA current and 0.15 atm pressure. The cathode and anode ionomer content was 10 wt % of the total solids in ink. The anode and cathode CCSs were coated with a top layer of Fumion FAA3 ionomer (10 wt % of total solid mass, fuel cell store) to reach 20 wt % ionomer content in both electrodes. To study the effect of ionomer (Nafion and Fumion), catalytic layers were prepared using only Nafion, only Fumion, and Nafion and Fumion (Nafion/ Fumion ratios of 0.35, 0.5, and 0.65) with cathode loading of 1 mg/cm^2^ and anode loading of 3 mg/cm^2^. The loading was then optimized to the performance of a state-of-the-art AEM electrolyzer that was prepared with cathode loading of 3 mg/cm^2^ and anode loading of 5 mg/cm^2^, as shown in the Supporting Information. The membrane electrode assembly (MEA) was formed by inserting the (Fumapem-3-PE-30) membrane between the cathode and anode electrodes as in Figure 1. The MEA was conditioned in 1 M KOH overnight to be exchanged to the hydroxideform. The measurements were performed in a modified Baltic cell connected to a Teflon tank. The setup used heaters and a peristaltic pump to inject electrolytes such as water (Milli-Q ultrapure water, 18.2 MΩ cm), 0.1, and 1 M KOH (≥85%, Sigma-Aldrich) at 50 °C through the AEMWE cell at a flow rate of 250 mL/min.

### Single-Cell Electrolyzer Testing

The electrochemical measurements of the single-cell electrolyzer were carried out using potentiostat/galvanostat (Biologic HCP-803 with a 20 A booster). The polarization curve is measured at constant current mode from 0 to 2 A/cm^2^ with 0.04 A/cm^2^ step every 30 s. To determine the cell resistance, the EIS was performed at a direct current (DC) of 0.2 A/cm^2^ with AC amplitude of ±5% of DC in the AC frequency range of 1 Hz to 100 kHz. For the durability test, the electrolyzer was held at 0.5 A/cm^2^ for 65 h in 0.1 M KOH. Galvanostatic EIS was performed every hour during the durability analysis at a current density of 0.5 A/cm^2^ in the AC frequency range of 1 Hz to 100 kHz with an amplitude of 25 mA/cm^2^. Post-mortem SEM and EDX have been carried out for the Ni–MoO_2_ nanosheet cathode electrode after the durability test.

## Results and discussion

### Structural Characterization

**Figure 2:**
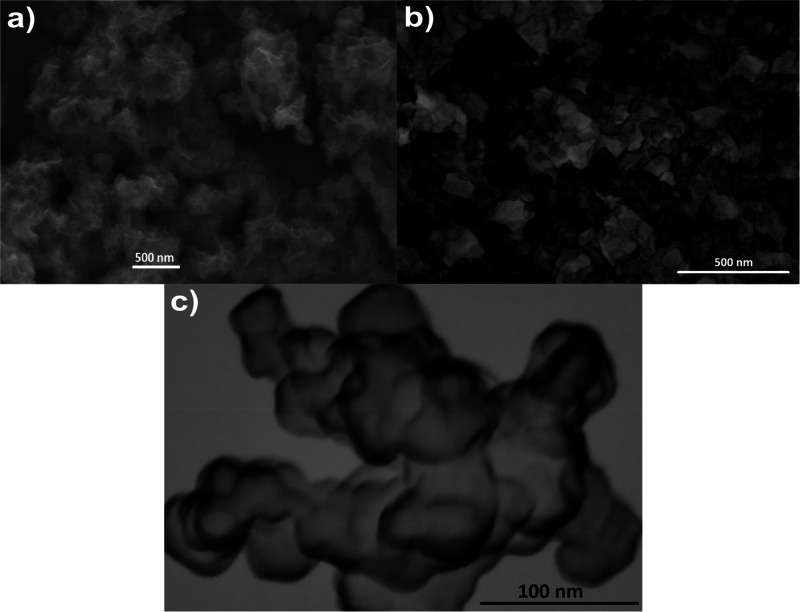
(a) SEM image and (b,c) STEM images at different magnifications of Ni–MoO_2_ nanosheets.

Figure 2 shows the morphological features of the Ni–MoO_2_ catalyst. The SEM and STEM images in Figure 2 show that the catalysts are composed of a network of interconnected nanosheets. High-resolution STEM images in Figure S1 confirmed this irregular nanosheet morphology. Catalysts synthesized by chemical reduction using sodium borohydride with similar morphological features have been termed nanocotton,^33^ nanosponges,^34^ or nanosheets.^35,36^ We will simply refer to catalysts like those in Figure 2 as nanosheets here, not to be confused with graphene or similarly well-structured single layers.

The formation of this particular morphology^37^ is believed to be due to hydrogen bubbles formed during the chemical reduction.^37^ The hydrogen bubbles will strongly disturb the structure during its making.^38^ The reduction reaction can be described as follows
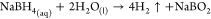
3
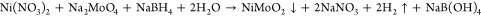
4An EDX analysis confirmed the presence of Ni, Mo, and O elements in the catalyst in Figure 2 and indicated that the catalyst is free of impurities from the synthesis precursors. The elemental composition of the metals is 78 atom % Ni and 22 atom % Mo as assessed by EDX (see Figure S2 in the Supporting Information for details), which is close to the nominal.

**Figure 3:**
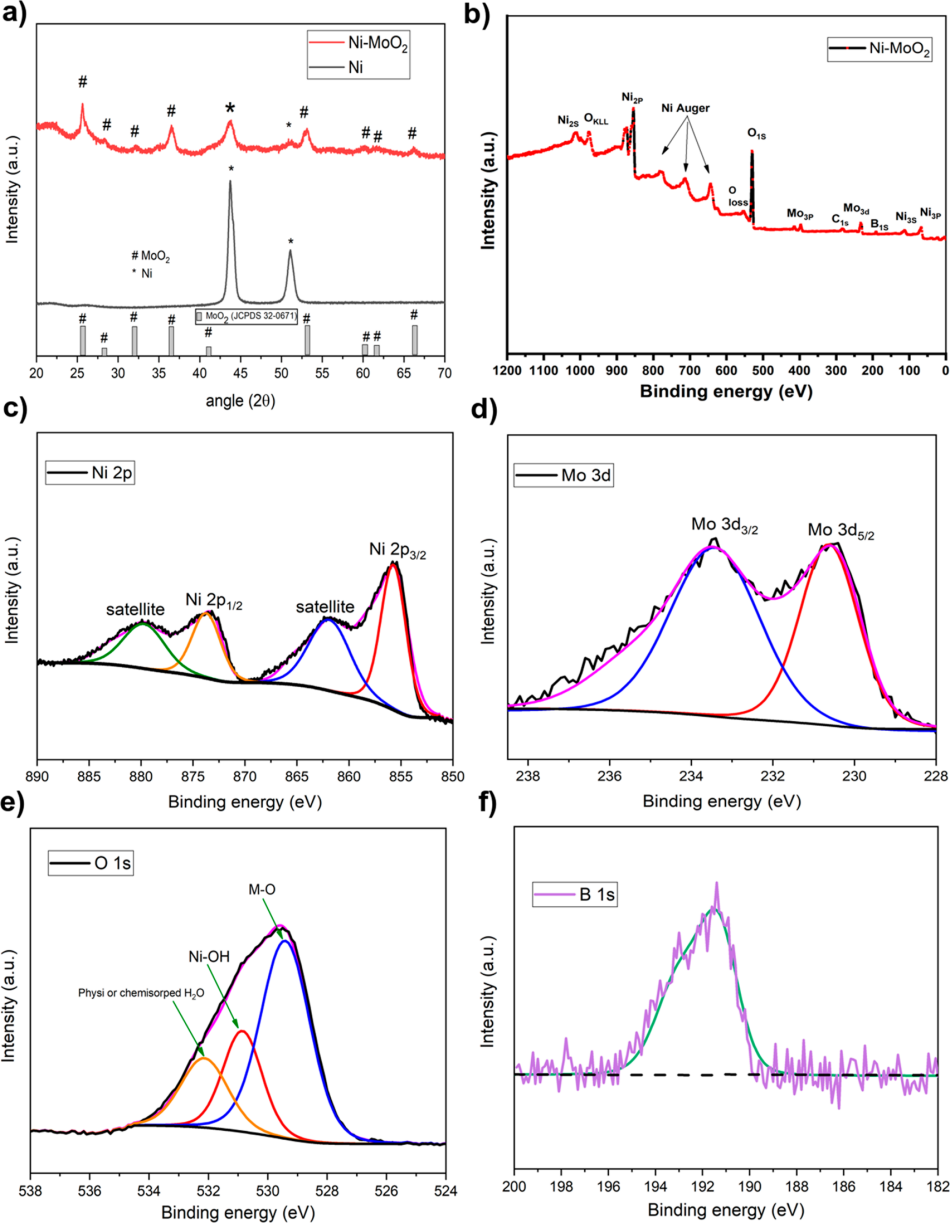
(a) XRD patterns of Ni–MoO_2_, and Ni nanosheets, (b) XPS survey spectrum of Ni–MoO_2_ nanosheets, and the corresponding high-resolution XPS spectrum of (c) Ni 2p, (d) Mo 3d, (e) O 1S, and (f) B 1s.

Figure 3a shows X-ray diffractograms (XRD) for Ni–MoO_2_ nanosheets. The XRD pattern of this annealed catalyst shows peaks at 2θ values of 44.3 and 51.6 indexed to the (111) and (200) planes of cubic Ni (JCPDS 04-0850), whereas peaks appearing at 25.8, 32.2, 36.6, 53.3, and 66.2 are assigned to monoclinic MoO_2_ (JCPDS 32-0671). The XRD result reveals that the catalyst has a hybrid structure consisting of Ni metal and MoO_2_.^39^

Figure 3b shows X-ray photoelectron spectroscopy (XPS) survey spectrum of Ni–MoO_2_ nanosheets catalyst. The spectrum shows that Ni, Mo, O, and B are present in the sample. The elemental fractions of Ni and Mo (with respect to the total metal) were found to be 77.21 and 22.79 respectively. The Ni 2p XPS spectra of the same catalyst are shown in Figure 3c. Two major peaks are observed with binding energies at 855.7 and 873.3 eV correlating to Ni 2p_3/2_ and Ni 2p_1/2_. The Ni 2p_3/2_ and Ni 2p_1/2_ energy separation of 17.6 eV is a characteristic of the Ni(OH)_2_ phase.^40^ The Mo 3d spectra are displayed in Figure 3d. The Mo 3d spectrum contains peaks at 230.5 (Mo^4+^ 3d5/2) and 233.6 (Mo^4+^ 3d3/2).^25,39^

An O 1s XPS spectrum is shown in Figure 3e of Ni–MoO_2_ nanosheets. Three peaks can be deconvoluted from the O 1s spectrum. The peak at 529.4 eV is related to the M–O (metal–oxygen) bond,^41^ the peak at 530.08 eV is attributed to the Ni–OH bond.^42^ The peak at 532.1 eV related to surface physi- or chemisorbed H_2_O.^43^ B 1s spectrum is displayed in Figure 3f. The XPS spectrum shows a peak at 191.5 eV binding energy (BE) indicating the presence of oxidized boron with no peaks for elemental boron.^44^

### Electrochemical Characterization

**Figure 4:**
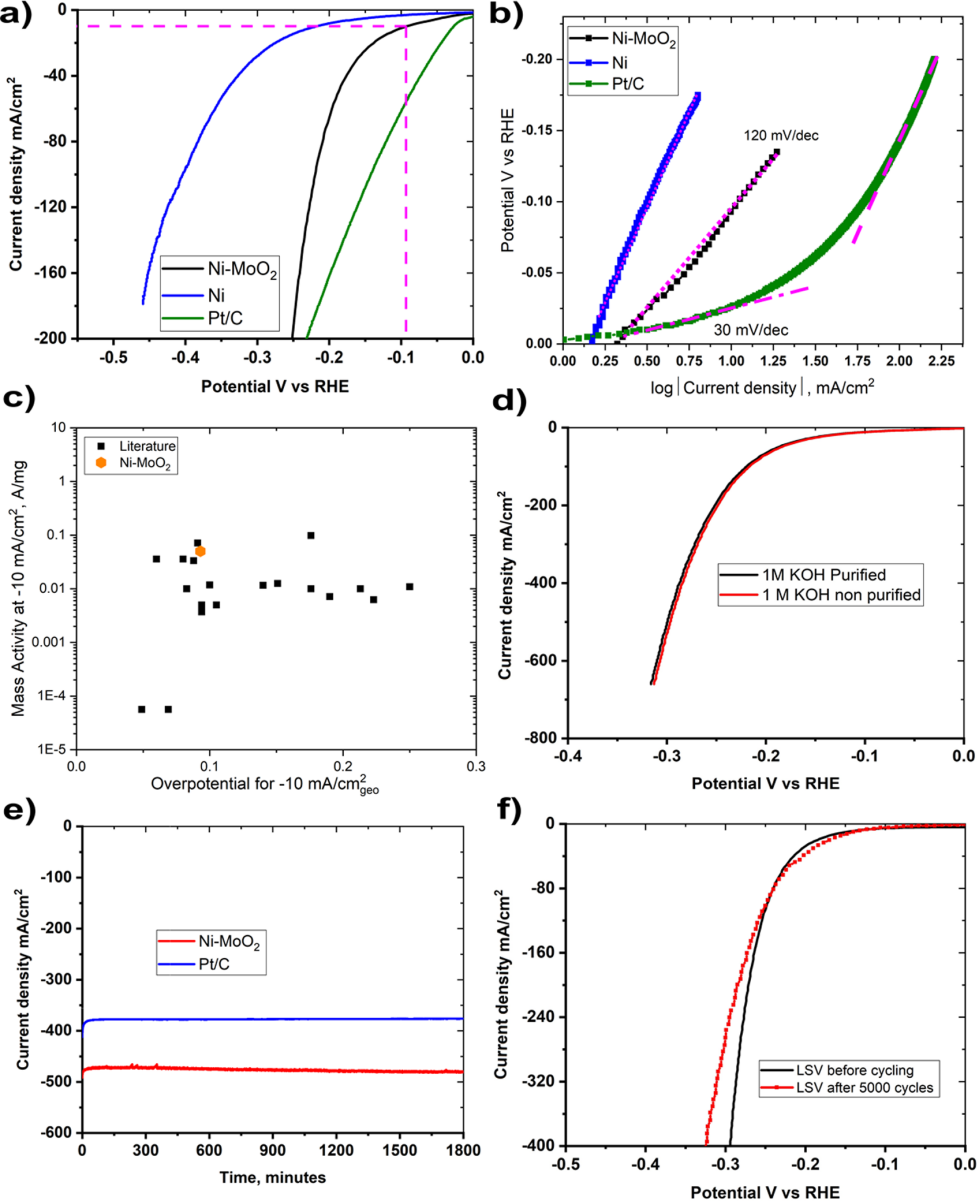
(a) LSVs and (b) Tafel plots for Ni–MoO_2_ and Ni nanosheets catalysts compared to Pt/C in 1 M KOH using Nafion ionomer, (c) Comparison of the mass activity of Ni–MoO_2_ nanosheets with literature data as summarized by Kibsgaard et al. Reprinted with permission from ref (28). Copyright 2019, Springer Nature. (d) LSVs of Ni–MoO_2_ nanosheets in purified and nonpurified 1 M KOH using Nafion ionomer, (e) chronoamperometry at −0.35 V for 1800 min (30 h) of Ni–MoO_2_ nanosheets and Pt/C in 1 M KOH using Nafion ionomer, (f) LSV of Ni–MoO_2_ nanosheets before and after 5000 cycles in 1 M KOH using Nafion ionomer.

Figure 4a shows HER linear-sweep voltammograms (LSVs) of Ni–MoO_2_ and Ni nanosheets in 1 M KOH. Figure 4a shows that Ni–MoO_2_ nanosheets achieved −10 mA/cm^2^ current density at an overpotential of −93 mV compared to −220 mV for Ni. Figure 4b shows plots of potential versus the logarithm of current density recorded for Ni–MoO_2_ nanosheets, Ni nanosheets, and Pt/C. The Tafel slope of Ni–MoO_2_ nanosheets is in the order of 120 mV/dec The mass activity of Ni–MoO_2_ is compared to several other HER catalysts for alkaline conditions in Figure 4c. The data for the catalysts to which we compare ours were taken from the recent review by Kibsgaard et al.^28^ Apparently, the mass activity of the Ni–MoO_2_ catalyst is among the best reported until now.

The effect of spurious iron in the solution is demonstrated in Figure 4d, which shows the LSVs of Ni–MoO_2_ nanosheets in purified and nonpurified KOH electrolytes. The activity of the Ni–MoO_2_ nanosheet catalysts for the HER is independent of whether or not the electrolyte is being purified. Despite previous reports that the presence of impurities in KOH electrolytes such as Fe increases the OER activity,^29^ we did not observe any such effect for HER activity of Ni–MoO_2_ nanosheets. The results in Figure 4d may serve as indirect proof for the absence of the effects of iron. (The KOH was purified using the same procedures as described by Trotochaud et al.^29^) Similar results were reported by Shalom et al.^45^ The activity of Ni–MoO_2_ nanosheets and Pt/C is also confirmed by cyclic voltammograms in Figure S3 (Supporting Information).

Figure 4e shows the stability of Ni–MoO_2_ nanosheets and Pt/C over 1800 min (30 h) in 1 M KOH. The Ni–MoO_2_ nanosheets maintain a stable performance of −475 ± 5 mA/cm^2^ at −0.35 V versus RHE without noticeable decay over time. Figure 4f shows the LSVs recorded for Ni–MoO_2_ nanosheets before and after the AST procedure. The Ni–MoO_2_ nanosheets show excellent cycling stability with a potential shift of 2, 10, and 15 mV at an HER current density of −100, −200, and −300 mA/cm^2^ respectively. The results in Figure 4 shows that Ni–MoO_2_ maintains a remarkable HER performance and stability with mass activity among the best reported until now.

Two reaction paths, viz. the Volmer–Heyrovsky or Volmer–Tafel schemes, are commonly used to express the overall HER reaction. The water electroreduction with hydrogen adsorption is represented by the Volmer reaction, while the electrochemical hydrogen desorption is represented by the Heyrovsky reaction and chemical desorption is involved in the Tafel reaction as in the equations below^46^
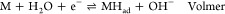
5
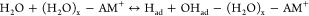
5a
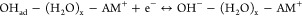
5b
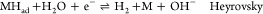
6
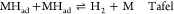
7A Tafel slope of 120 mV/dec is consistent with the Volmer reaction being rate-determining.^46^ In this reaction scheme, we have indicated the possible participation of cations, indicated as AM^+^, in the Volmer reaction. We have written the Volmer step as being composed of one step in which an OH_ad_–(H_2_O)–AM^+^ adduct is formed at the electrode (reaction reaction 5a), and a subsequent step eventually forming an OH^–^–(H_2_O)–AM^+^ adduct in the solution (reaction 5b). This picture of the Volmer reaction would be consistent with the 2B-theory proposed by E. Liu et al.^47,48^

The Tafel plots for the Pt/C electrode show two distinct slopes, which are commonly observed for Pt electrodes.^49,50^ At low overpotentials, the Tafel slope for Pt/C is around *b* ∼ 30 mV/dec, consistent with the Tafel reaction being the rate-determining step, following a fast initial Volmer discharge step. At high-overpotentials, the polarization curve for Pt/C has a Tafel slope approximately equal to *b* ∼ 120 mV/dec. A Tafel slope of 120 mV is expected for the Volmer step being rate-determining if the coverage of adsorbed hydrogen is high.^49,50^

**Figure 5:**
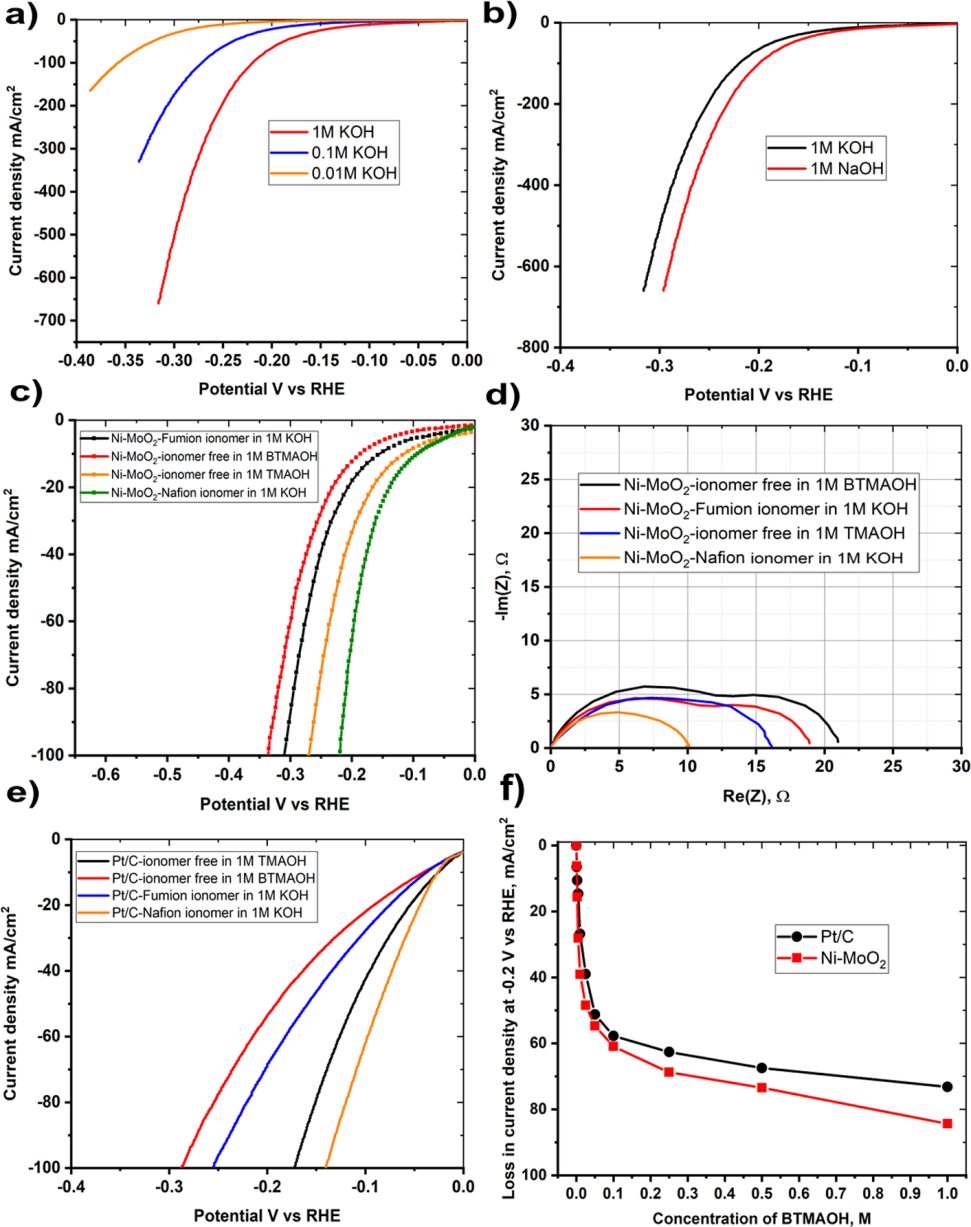
(a) LSVs of Ni–MoO_2_ nanosheets in 1, 0.1, and 0.01 M KOH concentrations, (b) LSVs of Ni–MoO_2_ nanosheets in 1 M KOH and 1 M NaOH, (c) LSVs Ni–MoO_2_ nanosheets in 1 M KOH using Fumion and Nafion ionomers and catalyst ionomer-free electrodes in organic cationic electrolytes (TMAOH and BTMAOH), (d) corresponding impedance complex plane plot at −0.25 V versus RHE at 1600 rpm rotation rate, (e) LSVs of Pt/C in 1 M KOH using Fumion and Nafion ionomers and catalyst ionomer-free electrodes in organic cationic electrolytes (TMAOH and BTMAOH), and (f) loss in current density at −0.2 V versus RHE versus BTMAOH concentration in [*Y* M KOH + (1 – *Y*) M BTMAOH] electrolyte for Ni–MoO_2_ nanosheets and Pt/C.

Figure 5 summarizes the effects of the ionomer and the composition of the electrolyte on the performance of Ni–MoO_2_ nanosheets catalyst. This includes a variation of the concentration of KOH (Figure 5a), the effects of the cation as represented by the substitution of KOH by NaOH (Figure 5b), the effects of organic cationic solutions benzyl trimethylammonium hydroxide (BTMAOH) and tetramethylammonium hydroxide (TMAOH), and the effect of the ionomer in the catalyst ink of the Ni–MoO_2_ nanosheets (Figure 5c,d) and Pt/C (Figure 5e). The influence of the addition of BTMAOH to KOH on the HER activity of Ni–MoO_2_ nanosheets and Pt/C is shown in Figure 5f. The BTMAOH and TMAOH organic cationic electrolytes were used to simulate the effects of the quaternary ammonium units in the ionomers since BTMAOH and TMAOH contain such units and therefore may serve as soluble analogues for the ionomer.

The most important factor appears to be the concentration of potassium hydroxide. Thus, going from 0.01 M KOH to 1 M KOH at −0.3 V increases the current by approximately 2 orders of magnitude, indicating a negative reaction order with respect to OH^–^ (Figure 5a). The cation also appears to affect the performance of the catalysts (Figure 5b). The HER activity for Ni–MoO_2_ nanosheets in 1 M NaOH is 50% higher than in 1 M KOH at −0.25 V versus RHE.

Next, the presence of quaternary ammonium units, either in the ionomer itself or as the solution analogues (BTMAOH or TMAOH), appear to play a significant role. The 1 M BTMAOH resulted in lower HER performance compared to TMAOH. The usage of 1 M BTMAOH leads to a decrease in the current at −0.15 V of approximately a factor of 2 with respect to the catalyst with Nafion ionomer in a solution of 1 M KOH, as is apparent both from the impedance data (Figure 5d) and the polarization curves of Ni–MoO_2_ (Figure 5c) and Pt/C (Figure 5e). For the same ionomer content, for Ni–MoO_2_ nanosheets and Pt/C, the application of the anion (Fumion) ionomer leads to lower HER activity than with Nafion ionomers in the thin film RDE electrodes.

The impedance–plane plot (obtained at −0.25 V versus RHE) with Nafion ionomer consists of a single semicircle, turning into two semicircles upon the addition of Fumion in the ink or using organic cationic electrolytes such as BTMAOH or TMAOH. The Nafion ionomer resulted in a lower low-frequency electrode resistance than the electrode with the anion exchange ionomer (Fumion) in the ink or if the electrolyte contained BTMAOH. The appearance of the second semicircle in the low-frequency regime has been proposed to be due to the adsorption of quaternary ammonium.^51^ The addition of BTMAOH to the KOH solution appears to be important even at very low concentrations as evidenced by the concentration dependence of the activity in Figure 5f. Figure 5f shows HER activity deterioration represented by current density loss at −0.2 V versus RHE versus BTMAOH concentration, Ni–MoO_2_ nanosheets, and a decrease of Pt/C HER activity as BTMAOH concentration increases. Ni–MoO_2_ nanosheets show a higher degree of activity loss with 84.4% of activity loss at −0.2 V compared to that of Pt/C (72.9%) in 1 M BTMAOH.

We previously derived a relation between reaction order and Tafel slope^4^ which suggests that with a Tafel slope of 120 mV the reaction rates should be independent of the OH^–^ concentration. However, experimentally the HER reaction rate of Ni–MoO_2_ nanosheets does depend on the concentration of KOH (Figure 5a). The Tafel slopes and the concentration dependence, therefore, indicate that rationalization of these would go beyond any simple microkinetic model, possibly by the incorporation of hydroxyl–water–cation adducts for the Volmer step, eqs 5a and 5b. According to the hard–soft acid–base (HSAB) theory, the metal cation is a Lewis hard acid and strongly binds with (OH^–^) which is a Lewis hard base, and weakly with the (OH_ad_) which is a Lewis soft base. The two side’s unbalanced binding energy of eq 5b induces the desorption of OH_ad_ to the electrolyte producing OH^–^. By increasing KOH concentration, enriching the abundance of the hydroxyl–water–alkali metal cation adduct will boost the Volmer step of HER.^47,52,48^ While such a mechanism may explain the effect of electrolyte cations, it may also have consequences for Tafel slopes.

The HER activity increases when replacing K^+^ with Na^+^ in hydroxide solution (Figure 5b) and suggests that the effect of the cation type through the formation of hydroxyl–water–cation adducts^47,52^ would lead to a much more moderate change than what is observed in Figure 5a with changing KOH concentration.

**Figure 6:**
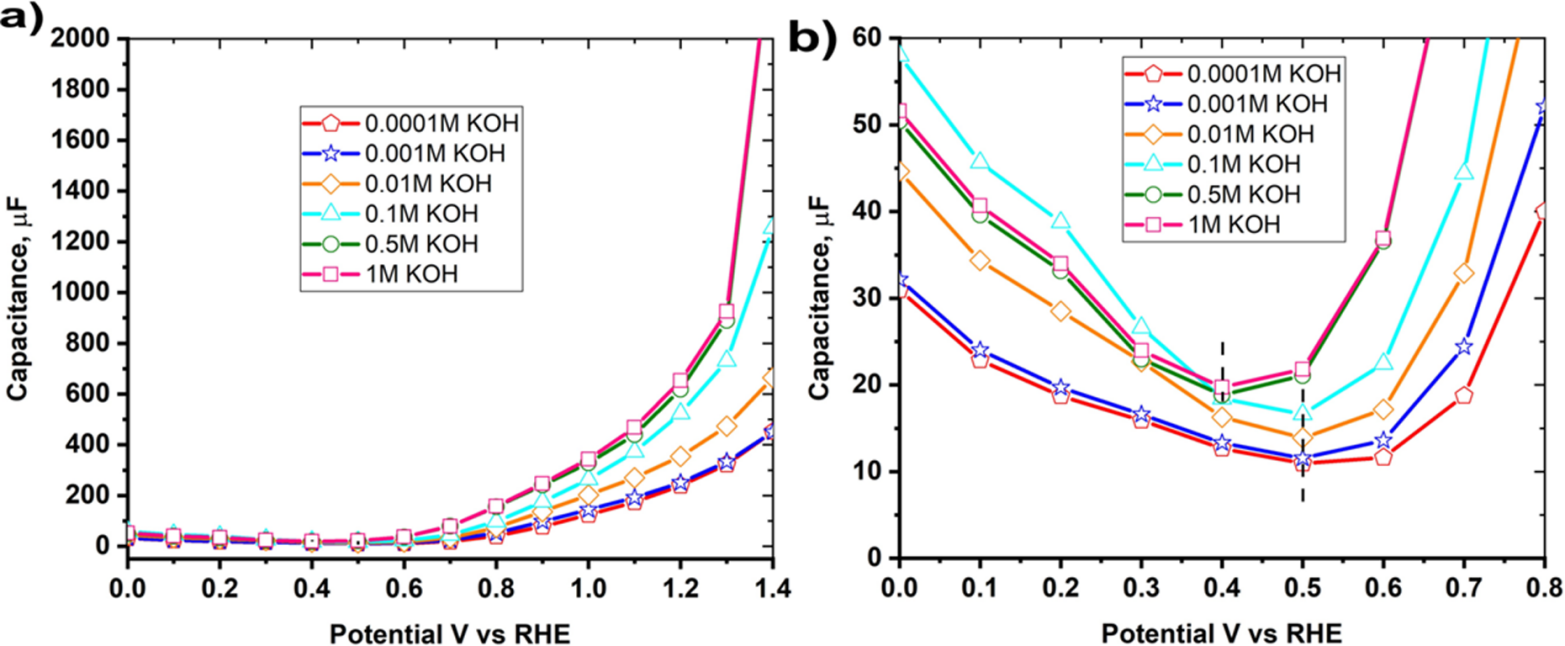
(a) Capacitance–potential curves gathered from the impedance measurements of Ni–MoO_2_ nanosheets in various KOH concentrations (1 M to 0.1 mM). (b) Magnified view of the capacitance–potential curves displaying the changes in potential for capacitance minimum at different concentrations of KOH electrolyte.

The effect of the KOH concentration in Figure 5a may be also related to the pzc of the electrode. As commented above, in this work we have assumed that the pzfc can be inferred from the potentials at which the capacitance is minimum. Figure 6 shows capacitance–potential curves for Ni–MoO_2_ nanosheets in various KOH electrolyte concentrations at 10 Hz. A magnified view is offered in Figure 6b. The capacitance minimum is at 0.5 V versus RHE for 0.1 mM to 0.1 M KOH but shifted to lower potentials at 0.4 V versus RHE in 0.5 and 1 M KOH. The capacitance was measured using electrochemical impedance spectroscopy from −0.1 to 1.4 V versus RHE applying frequencies from 10 Hz to 1 kHz with a 5 mV perturbation amplitude. The potential for the capacitance minimum found in this work (0.4 V versus RHE or (−0.5 V versus Hg/HgO)) in 1 M KOH for Ni–MoO_2_ nanosheets is comparable to the values for nickel (−0.47 versus Hg/HgO) in 8 M KOH reported by Gagona et al.^32^ The potential of the capacity minimum for Ni–MoO_2_ nanosheets at 10^–4^ M KOH (0.5 V versus RHE) is close to the nickel pzc value of −0.25 V versus NHE (0.4 V versus RHE) in 10^–4^ M NaOH reported by Bockris et al.^21^

To check the accuracy of capacitance minimum, capacitance minimum measurements were carried out with different intervals of 100 and 25 mV and both show the capacitance minimum in 1 M KOH at 0.4 V versus RHE as in Figure S4a. The capacitance minimum is shifted to positive potential values when the TMAOH electrolyte is used (Figure S4b). A similar method for measuring the capacitance minimum has previously been used in the literature.^31,21,32^ The capacitance minimum is constant for all applied frequencies (Figure S5). The effect of frequency on capacitance minimum was studied also by Bockris et al., and they found that the capacitance minimum is independent of frequency.^21^

According to pzfc theory, shifting the pzfc toward a negative potential (closer to HER onset potential) leads to the facilitation of the O–H scission process by lowering the charge transfer barrier through the water network. This reduces the interfacial electric field at the potential range for HER resulting in more facile interfacial water network reorganization and softens the double-layer to promote OH^–^ and H^+^ transport for alkaline and acid electrolytes, respectively. In both cases, this facilitates the Volmer step and enhances the rate of the HER.^22^

When the pzfc is shifted to positive potentials, this increases the strength of the electric field, which leads to the accumulation of ions, solvent ordering, and orientation of dipolar species (water); this increases the water reorganization energy, makes the double layer more rigid, and restricts the transport of charged reactants/products such as OH^–^, corresponding to a higher energy barrier of Volmer (or Heyrovsky) step causing a lower HER activity.^22,23^

In this work, when KOH concentration increased from 0.1 mM to 1 M KOH, the pzfc value of Ni–MoO_2_ nanosheets shifted negatively to lower potential values on the RHE scale. The fact that the capacitance minimum in Figure 6b decreases with increasing KOH concentration appears to support the interpretation of the closer pzfc to the HER equilibrium potential, the higher the activity for the HER. The pzfc negative shift suggests a reduction in the interfacial electric field and softening of the double-layer to promote OH^–^ transport which leads to higher kinetics of the Volmer step and HER activity.^23^ However, at the potentials above 1 V in Figure 6a, the capacitance may involve oxidation of Ni(OH)_2_ to NiOOH.^53^

The charge of the electrode (pzfc) is crucial also to explain the interaction with the cationic moieties (positively charged ammonium units) of the ionomer. If the capacitance minimum in Figure 6 is taken to reflect the pzfc for the electrode, the electrode is negatively charged during the HER. The positively charged quaternary ammonium (QA^+^) species will be adsorbed/attracted to the electrode surface and thus play a role in blocking the electrode. When a negative potential is applied, the quaternary ammonium (QA^+^) from organic electrolytes such as BTMAOH or TMAOH solution or anion ionomer is adsorbed on the catalyst surface, forming a compact inner layer of positive charges and blocking the surface from access to the electrolyte and the HER, as demonstrated by the results in Figure 5.^54,55^ The capacitance minimum is shifted to positive potential values when TMAOH electrolyte is used (Figure S4b). The pzfc shift to more positive potentials increases the magnitude of the (negative) electric field at the electrode surface (i.e pointing out of the electrode and into the electrolyte) at the potential of the HER. This increase in the magnitude of the electric field tends to make water reorganization less facile and may in fact at very high electric fields result in a solid-state like structure resembling ice.^56−58^ A more rigid water layer adjacent to the electrode would raise the energy barrier for the transport of hydroxide ions through the layer, which is required for the HER to proceed. Thus, a positive shift in the pzfc results in a lower HER activity.^23^

**Figure 7:**
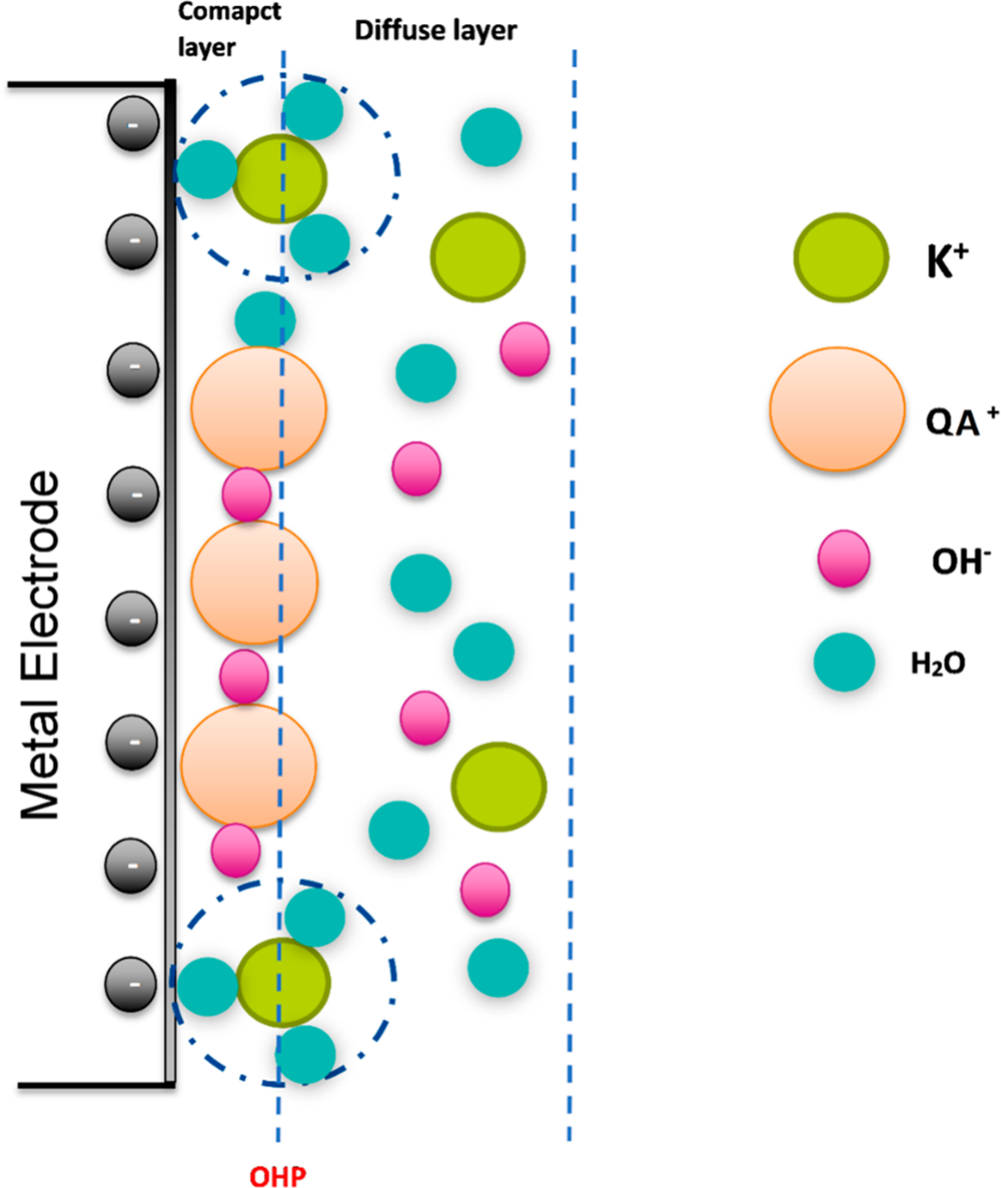
Schematic of the double layer during HER in the presence of quaternary ammonium species and KOH electrolyte.

The blocking of the surface site also depends on the moiety to which the quaternary ammonium is attached,^59^ that is, the HER activity in BTMAOH than in TMAOH. The BTMAOH, therefore, is more significant in blocking active sites. DFT results by McCrum et al. suggested that this effect is due to the interaction between the benzyl group and electrode surface.^59^ TMAOH has been found to inhibit the HER by cation–hydroxide–water coadsorption under alkaline conditions.^51,60,61^ Infrared reflection absorption spectroscopy (IRRAS) studies indicate that the adsorption of tetramethylammonium (TMA^+^) cation causes hydroxide and water coadsorption on the surface of Pt catalyst with higher hydroxide concentration compared to water.^51,60,61^ The mobile QA^+^ cations from organic electrolytes can form a more compact double layer due to their mobility, and hence the blockage in the case of organic electrolytes will be in a similar manner as the anion ionomer as found in this study. Our model for the ionomer interaction is summarized in Figure 7.

This work shows the importance of the catalyst interaction with the cationic group and polymer backbone of anion ionomers when optimizing active electrodes.^59^ The work here agrees with previous reports that ionomer with fewer phenyl moieties, smaller alkyl chain length can minimize the negative impact of ionomer on HER activity.^59^

**Figure 8:**
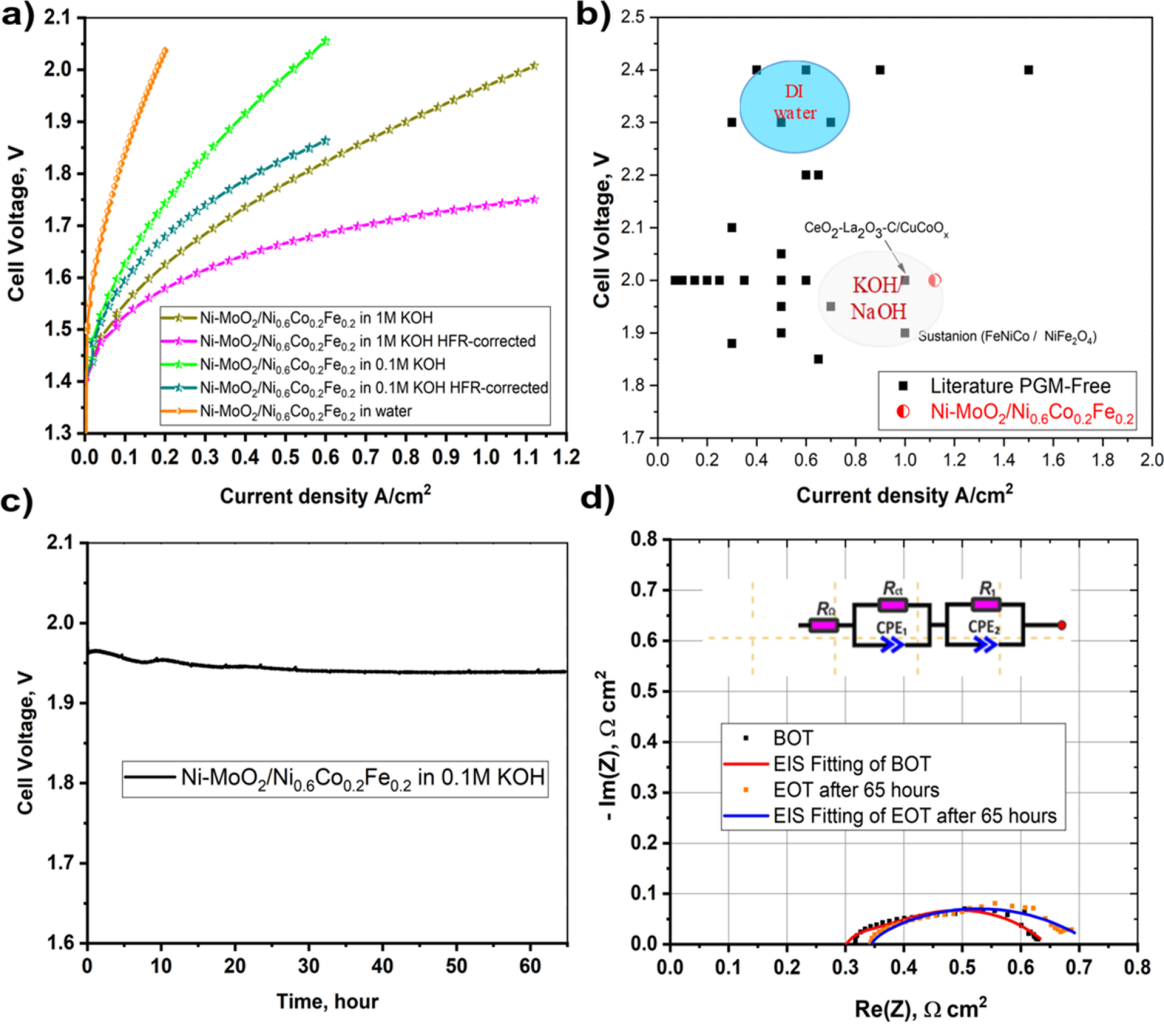
(a) Raw and HFR corrected polarization curves of Ni–MoO_2_ nanosheets/Ni_0.6_Co_0.2_Fe_0.2_ AEM electrolyzer in various electrolytes. (b) The Ni–MoO_2_ nanosheets/Ni_0.6_Co_0.2_Fe_0.2_ electrolyzer activity comparison with literature data. Published with permission from ref (62). Copyright 2019, Royal Society of Chemistry. (c) The AEM electrolyzer stability profile for 65 h, (d) EIS complex plane plot at the beginning and end of stability test (BOT and EOT) in 0.1 M KOH for 65 h recorded at 0.5 A/cm^2^ of Ni–MoO_2_ nanosheets/Ni_0.6_Co_0.2_Fe_0.2_ AEM electrolyzer.

We finally wish to point out the practical utility of the results in single-cell electrolyzer testing. Figure 8 shows the performance data for a full non-PGM AEM electrolyzer with a cathode catalytic layer of Ni–MoO_2_ nanosheet catalysts. The results for catalytic layers containing different Fumion and Nafion ratios showed that layers containing Nafion gave the best electrolyzer performance in 1 M KOH. (Details are given in Figure S6a.) The loading was optimized to give the state-of-the-art AEM electrolyzer performance (Figure S6b). The anode catalytic layers were prepared similarly and contained a laboratory-synthesized and optimized Ni_0.6_Co_0.2_Fe_0.2_ catalyst with a loading of 5 mg/cm^2^. The state-of-the-art electrolyzer catalyst layers was prepared with a cathode catalyst loading of 3 mg/cm^2^, and the layer contained 10 wt % Nafion with a thin top layer of anion Fumion ionomer (10 wt % of total solid mass). The catalytic layers were furnished with a top layer of an anion-conducting Fumion polymer to improve ionic conductivity at low KOH concentrations (Figure S7).

Figure 8a shows the polarization curves for the AEM electrolyzer with circulation electrolytes of 0.1 M KOH and 1 M KOH at 50 °C. Both the raw data and high-frequency resistance (HFR)-compensated data are included. We achieved a current density of 1.15 A/cm^2^ at 2 V cell voltage in 1 M KOH while achieving 0.65 A/cm^2^ at the same cell voltage in 0.1 M KOH at 50 °C. The electrolyzer performance showed good reproducibility in 1 M KOH, c.f. Figure S8. Figure 8b illustrates the performance of the cell with Ni–MoO_2_ and Ni_0.6_Co_0.2_Fe_0.2_ catalysts with respect to other values for non-PGM catalysts from the literature.^62^ With catalysts tuned as above, an AEM cell with Ni–MoO_2_ and Ni_0.6_Co_0.2_Fe_0.2_ catalysts is obtained that appears to be among the best reported so far, demonstrating a current density of 1.15 A/cm^2^ at 2 V. The performance with the 1 M KOH is significantly better than that in 0.1 M KOH. Also, the difference between the *iR*-compensated curves with those that were not corrected for ohmic losses shows that ohmic losses are an important part of the total losses. Circulating water not containing any KOH in the AEM electrolyzer resulted in a performance of merely 0.2 A/cm^2^ at 2 V. The electrolyzer performance using 0.1 and 1 M KOH is much better than water only, which is consistent with recent literature by Kraglund et al.,^62^ Henkensmeier et al.,^63^ Miller et al.,^64^ Park et al.,^65^ Lim et al.,^66^ and Vincent et al.^6^ Recently, Ayers et al.^67^ referred in their recent review to the change in performance between water and KOH to catalyst–ionomer–electrolyte interaction. Using the same approach for the catalytic layer fabrication, a cell with a Pt–C/Ir MEA achieved 2.6 A/cm^2^ at 2 V in 1 M KOH at 50 °C, which is comparable to the best AEM electrolysis performance using PGM catalysts, see Figure S9.

The EIS during electrolyzer testing was performed to estimate the ohmic resistance and allow for a separation of this resistance from other contributions to the AEM water electrolysis cell voltage. The cell resistance is evaluated from the high-frequency resistance (HFR) implied from the intercept with the real axis.^68^Figure S10 displays the EIS complex-plane plot obtained at 0.2 A/cm^2^ for Ni–MoO_2_/Ni_0.6_Co_0.2_Fe_0.2_ cells in 0.1 and 1 M KOH. For 1 M KOH, the Ni–MoO_2_ cell has an HFR of 0.2335 Ω cm^2^ compared to 0.3175 Ω cm^2^ in 0.1 M KOH. Decreasing KOH concentration from 1 to 0.1 M KOH rises the HFR, which proposes insufficient membrane conductivity.^26^ The EIS complex–plane plots that contain two overlapping and depressed semicircles with the arc at low-frequency is suggested to correspond to mass transfer limitations.^69,68^ We display the equivalent circuit to fit the EIS data in Figure S10. The equivalent circuit to which the data were fitted consisted of two *R*-CPE parallel combinations in series with a resistor. The fitting parameters are summarized in Table S1. *R*_Ω_ correlates with the cell ohmic resistance (membranes, electrodes, and current collectors). *R*_ct_ represents the cathode and anode resistance for charge transfer and may also include other contributions such as adsorption of intermediates. CPE_1_ is a constant phase element that we take to represent the capacitive charging of a rough electrode. The equivalent circuit has an extra *R*-CPE combination, where CPE_2_ and *R*_1_ are proposed to represent the formation of bubbles and mass transport at electrode–electrolyte interface.^69^

Figure 8c shows the AEM electrolyzer cell voltage in 0.1 M KOH for 65 h at 0.5 A/cm^2^. The voltage decreases from an initial 1.96 to 1.94 V with the major change in the voltage appearing during the first 30 h. During the last 20 h, there is no significant change in voltage with time. This result indicates a robust electrode that remained intact throughout the test and sets a new steady-state benchmark for accumulated hours on a non-PGM cell. Images of the MEA after the stability test are shown in the Supporting Information (Figure S11). Photographs collected after the durability test indicated that the MEA was still in good condition without visible voids or other signs of degradation. Corresponding elemental maps obtained by energy-dispersive X-ray spectroscopy (EDX) showed a homogeneous coverage and a nickel content of 88 atom % and molybdenum content of 12 atom %, which are reasonably close to those expected for the pristine Ni–MoO_2_ catalyst (Figure S12).

Figure 8d shows EIS data recorded for a cell immediately after cell assembly and data recorded after 65 h of operation in 0.1 M KOH. The complex impedance-plane plots for data recorded at a current density of 0.5 A/cm^2^ for the Ni–MoO_2_/Ni_0.6_Co_0.2_Fe_0.2_ cells before and after stability test form depressed, somewhat elongated semicircles and indicate two partly overlapping time-constants. Also, these data were fitted to an equivalent circuit containing two *R*-CPE parallel combinations in series with a resistor (see insert of Figure 8d). The fitting parameters are summarized in Table S2. The high-frequency intercept of the EIS complex plane plot with the real axis was taken to represent the cell (ohmic) resistance.^68^ The cell resistance of the electrolyzer increased from 0.3025 to 0.3382 Ω cm^2^ which corresponds to an 11.4% increase in HFR after 65 h. The total polarization resistance deduced from the semicircle diameter increased only slightly from 0.34 to 0.365 Ω cm^2^ after 65 h. The stability of the polarization resistance for the cell indicates that catalyst layer degradation is negligible in the cell over time.

Modern industrial alkaline electrolyzers operate at a current density of 0.45 A/cm^2^ at 1.7–2.1 V cell voltage^70^ while our Ni–MoO_2_/Ni_0.6_Co_0.2_Fe_0.2_ AEM electrolyzer achieves twice the current density in the same potential range. Therefore, Ni–MoO_2_ nanosheets’ activity and stability allow for active and cheap electrodes for AEM water electrolysis.^70^

## Conclusions

The HER activity of Ni–MoO_2_ nanosheets depends on electrolyte organic cation type (TMAOH, BTMAOH) and concentration, ionomer chemistry (Fumion, Nafion), and electrolyte inorganic cation (Na^+^, K^+^) and concentration. The HER activity increases when replacing K^+^ with Na^+^ in 1 M hydroxide solution. The capacitance minimum, which we relate to the pzfc, was negatively shifted when the KOH concentration increased from 0.1 mM to 1 M KOH. This suggests a reduction in the interfacial electric field, softening of the double-layer to facilitate the OH^–^ transport, leading to higher HER performance. The effect of ionomer–catalyst interaction can also be rationalized on the same basis; since the potential of capacitance minimum indicates a negatively charged catalyst surface in the HER potential range, the surface will attract the cationic moieties in the anion-conducting ionomer with adverse consequences for the catalytic activity. Anion Fumion ionomer and electrolytes with organic cations with benzyl group adsorption (BTMAOH) lead to lower HER performance in comparison with TMAOH and Nafion. In a full non-PGM AEM electrolyzer with Ni–MoO_2_ nanosheets electrode as the cathode [using Nafion ionomer ink and a top layer of Fumion anion exchange ionomer], the electrolyzer achieved a current density of 1.15 A/cm^2^ at 2 V cell voltage in 1 M KOH at 50 °C with outstanding durability in 0.1 M KOH for 65 h at 0.5 A/cm^2^.

## Supporting Information Available

STEM images and EDX spectrum; electrochemical characterization of catalysts in rotating disk electrode configuration and single-cell electrolyzer; postmortem analysis of cathode catalyst layer (PDF)
