# Supplementary material for: Tuning Ni–MoO2 Catalyst–Ionomer and Electrolyte Interaction for Water Electrolyzers with Anion Exchange Membranes
Source: ACS Appl Energy Mater. 2021 Mar 23;4(4):3327–40. doi: 10.1021/acsaem.0c03072 (PMC8159162; doi:10.1021/acsaem.0c03072)
Supplement: Supplementary file 1 — ae0c03072_si_001.pdf [file ae0c03072_si_001.pdf]

Supporting Information

Tuning Ni-MoO_2 Catalyst–Ionomer and Electrolyte Interaction for Water Electrolyzers with Anion Exchange Membranes

Alaa Y. Faid[1,], Alejandro Oyarce Barnett[2,3], Frode Seland[1], and Svein Sunde [1].*

[1]Department of Materials Science and Engineering, Norwegian University of Science and Technology, Trondheim, Norway

[2]SINTEF Industry, New Energy Solutions Department, Trondheim, Norway

[3]Department of Energy and Process Engineering, Norwegian University of Science and Technology

*** Corresponding Author:** alaa.faid@ntnu.no

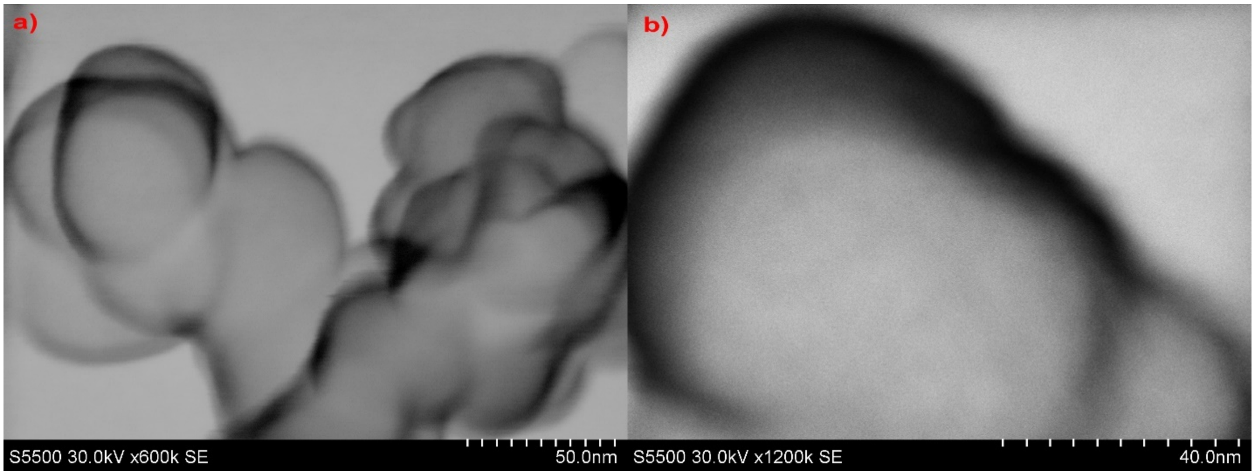

Figure S1: a) and b) STEM images of Ni-MoO_2 nanosheets.

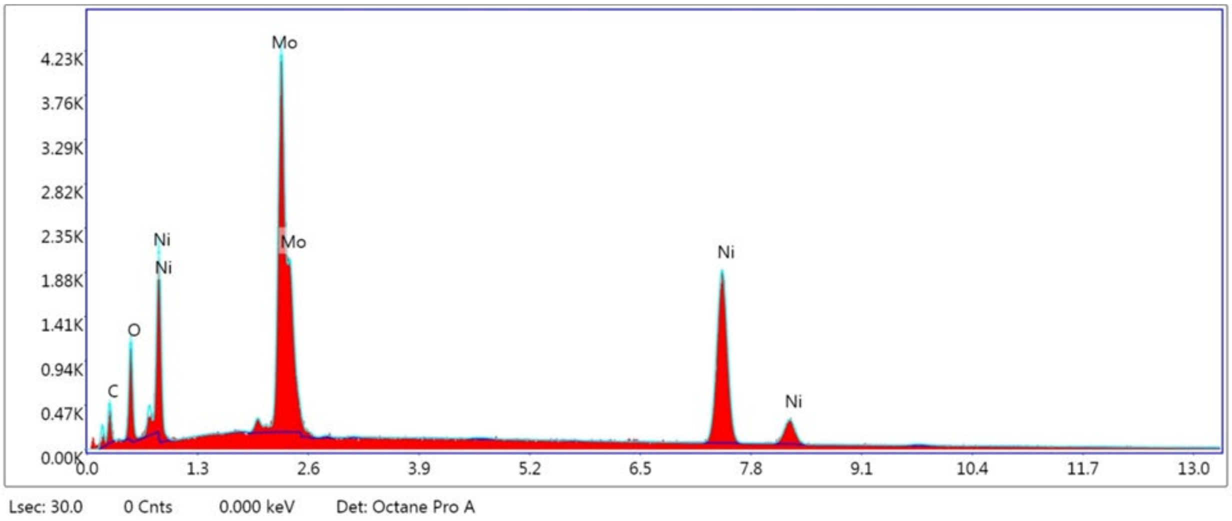

Figure S2: EDX spectrum of Ni-MoO_2 nanosheets.

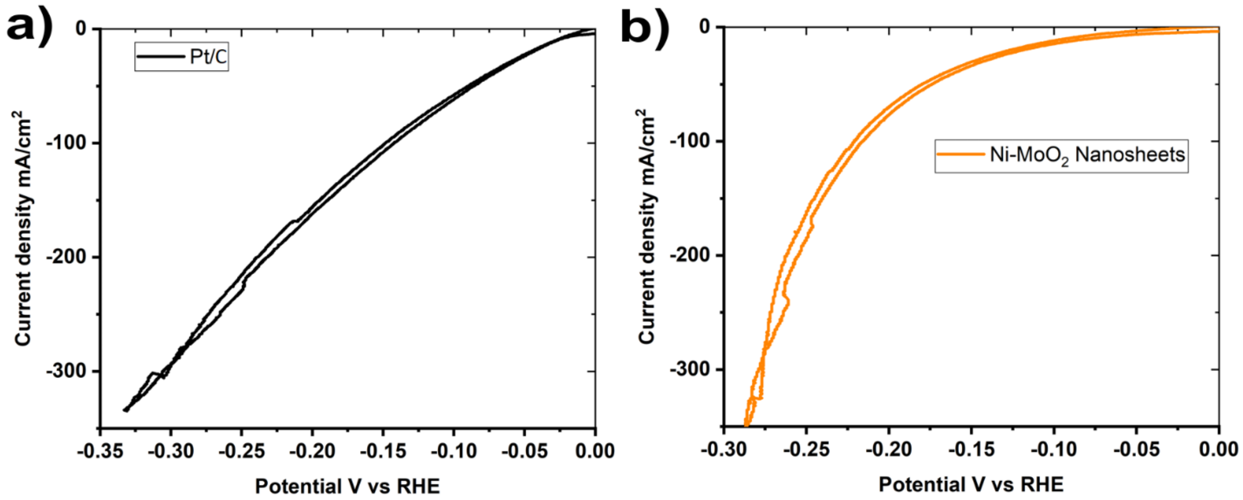

Figure S3: Cyclic voltammetry of a)Pt/C and b) Ni-MoO$_2$ nanosheets in 1M KOH.

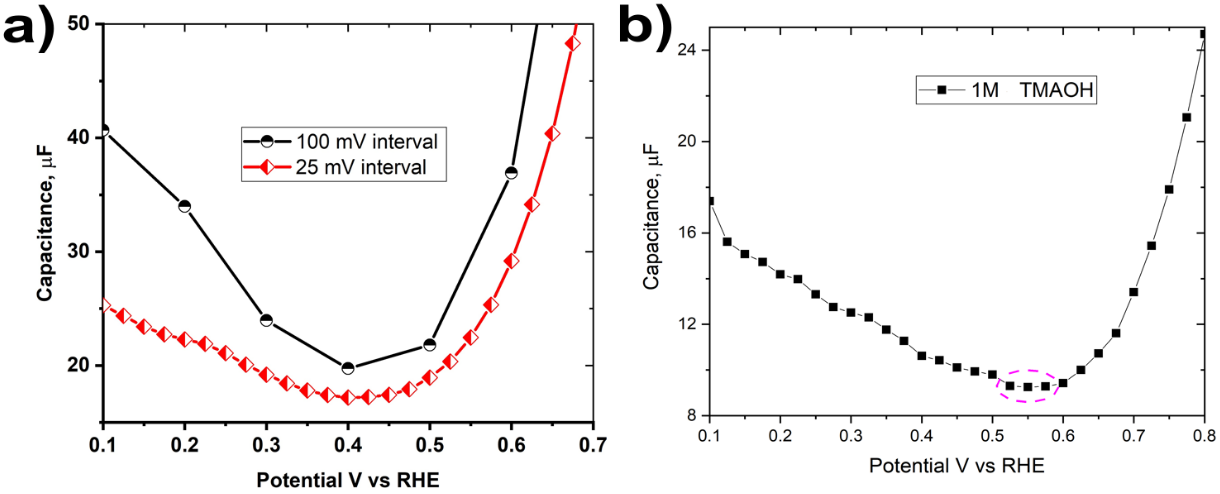

Figure S4: Capacitance-potential relation for Ni-MoO_2 nanosheets in a) 1 M KOH at 10 Hz applied frequency with 100 and 25 mV potential intervals, b) 1M TMAOH.

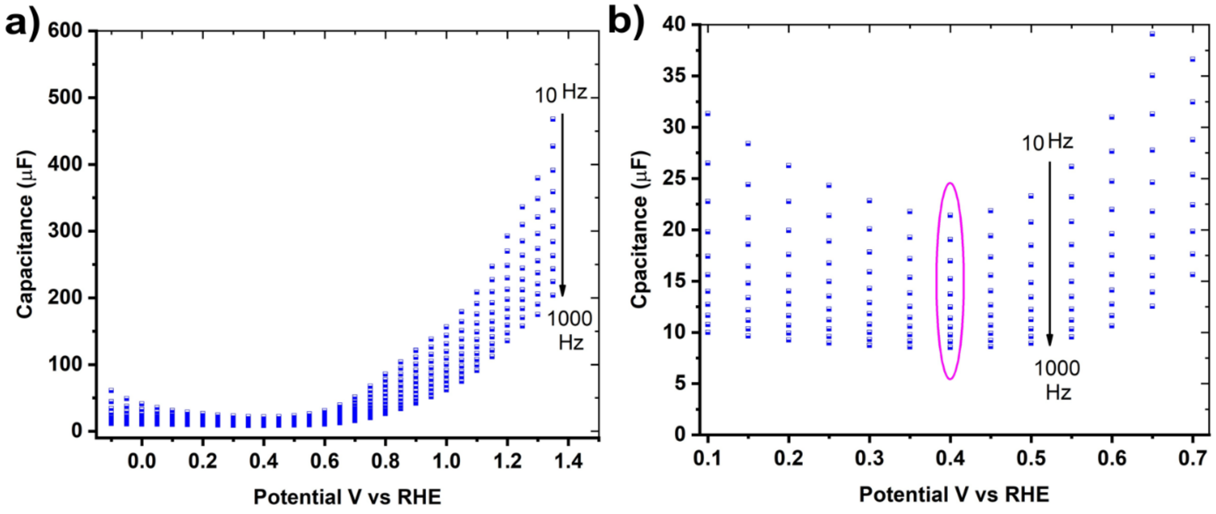

Figure S5: a) Full scale and b) Enlarged capacitance potential relation for Ni-MoO_2 nanosheets in 1M KOH at various applied frequencies.

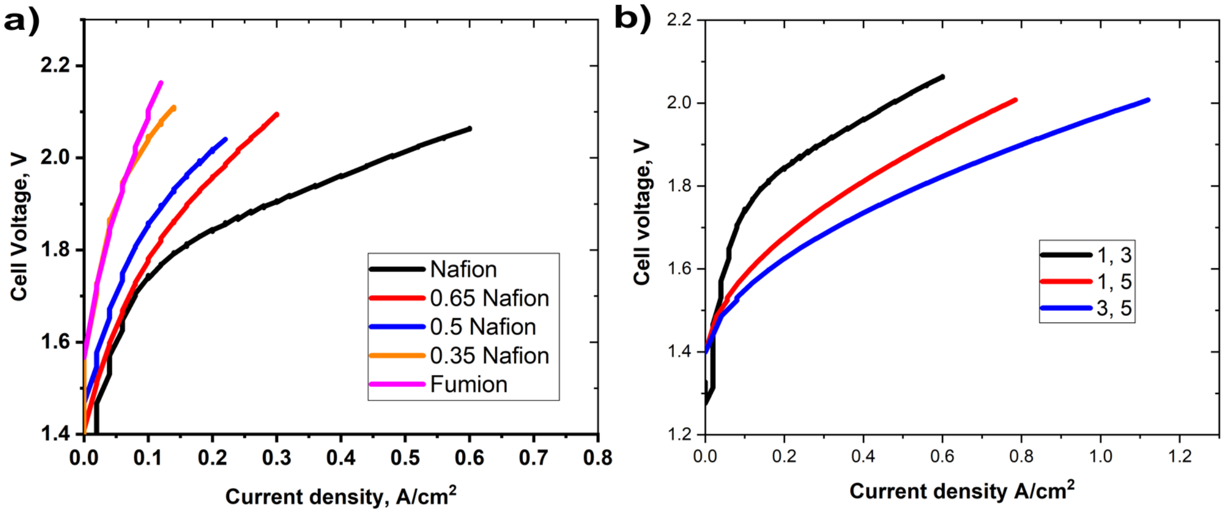

Figure S6: a) Polarization curves with various ionomer content of [Y Nafion + 1-Y Fumion ionomer] % in catalytic layers at 1 mg/cm^2 Ni-MoO_2 and 3 mg/cm^2 $Ni_{0.6}Co_{0.2}Fe_{0.2}$ in 1 M KOH, b) for the same ionomer content (10 wt % Nafion), the loading was optimized as (1, 3) , (1, 5), and (3, 5) mg/cm^2 for cathode and anode respectively.

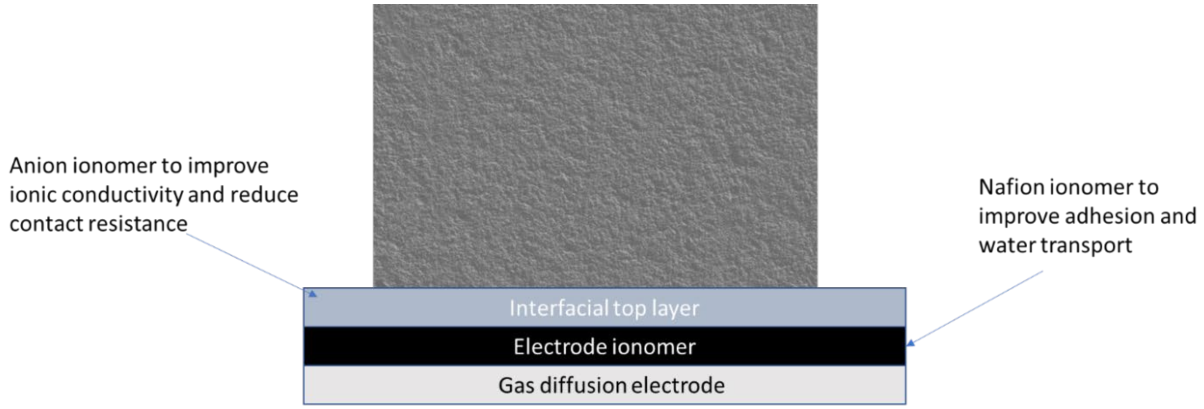

Figure S7: Schematic of ionomer type role in the electrode and as a top layer, figure reproduced from Ayers et al. [1]

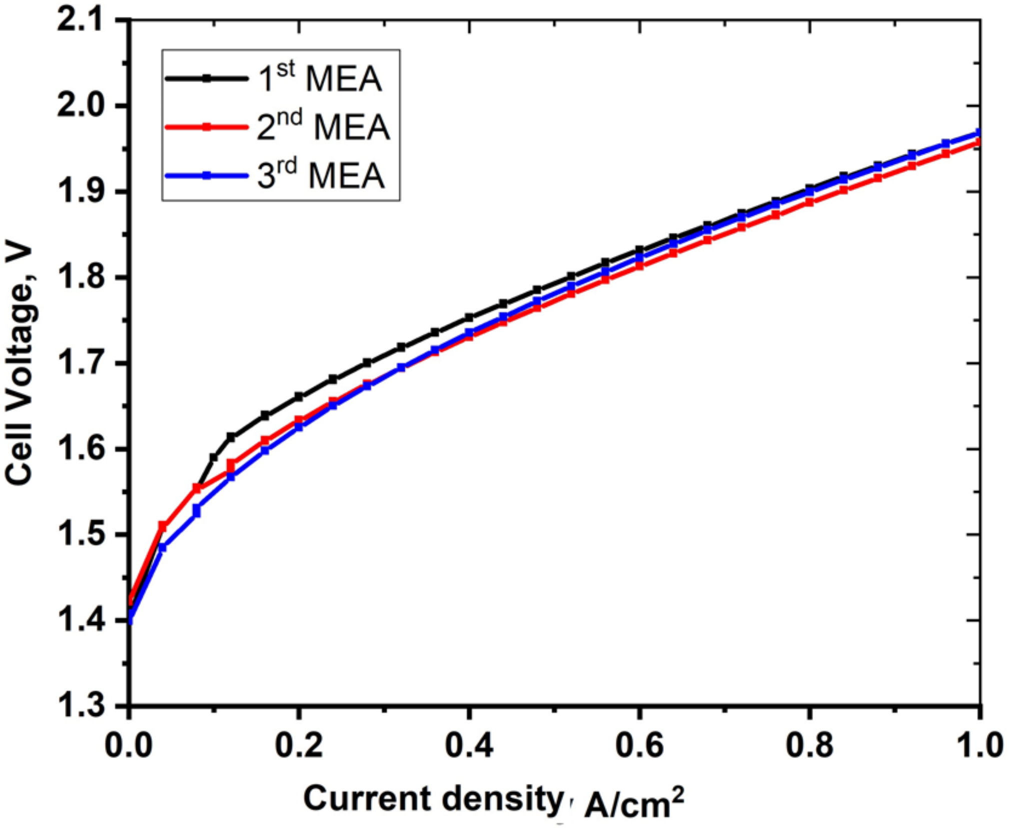

Figure S8: Reproducibility of three different Ni-MoO_2/ $Ni_{0.6}Co_{0.2}Fe_{0.2}$ MEAs in 1 M KOH.

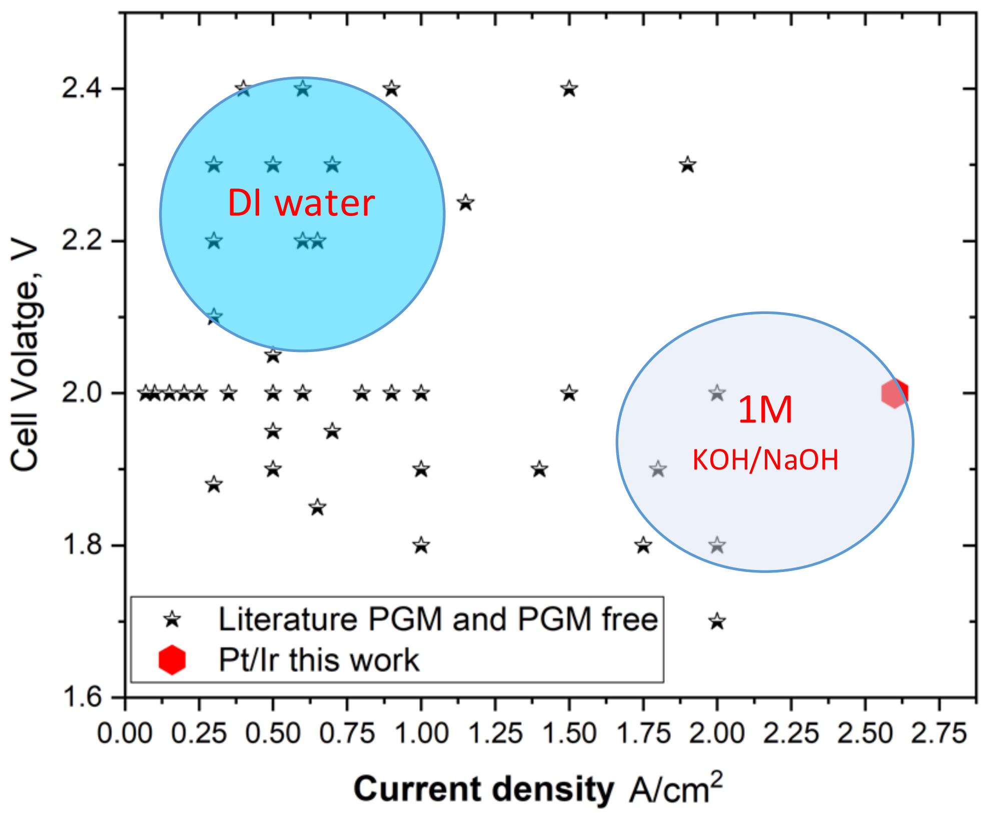

Figure S9: AEM (Pt/Ir) electrolyzer activity of comparison with literature with data reproduced from Kraglund et al.[2] published with permission from the Royal Society of Chemistry.

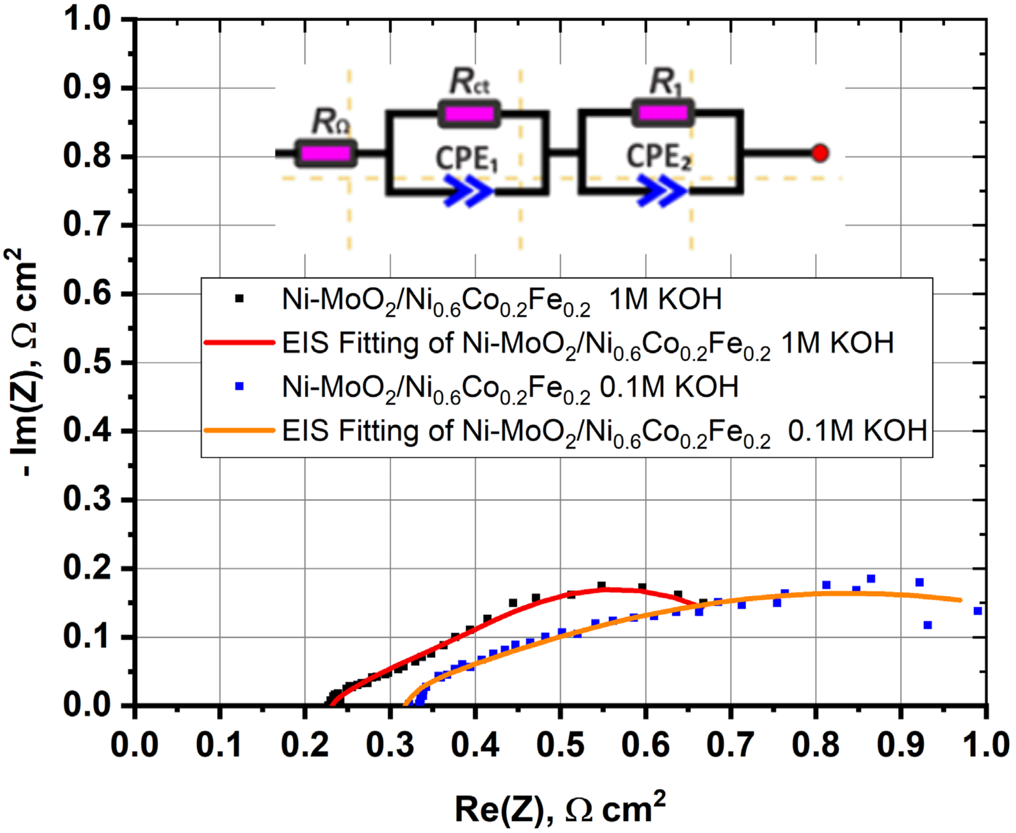

Figure S10: Electrochemical impedance spectroscopy (EIS) complex plane plot of Ni-MoO$_2$ / Ni$_{0.6}$Co$_{0.2}$Fe$_{02}$ in 1 and 0.1 M KOH.

Table S1: Parameters extracted from impedance data fitting for Ni-MoO_2/ $Ni_{0.6}Co_{0.2}Fe_{0.2}$ electrolysis cells.

Impedance fitting	Ni-MoO_2/NiCoFe 1 M KOH	Ni-MoO_2/NiCoFe 0.1 M KOH
R_{Ω}	0.2335	0.3175
Q1	183.625	153.675
A1	0.438	0.46
R_{ct}	0.53475	0.6950
Q2	19.48	16.19
A2	0.95	0.99
R1	0.07325	0.15225

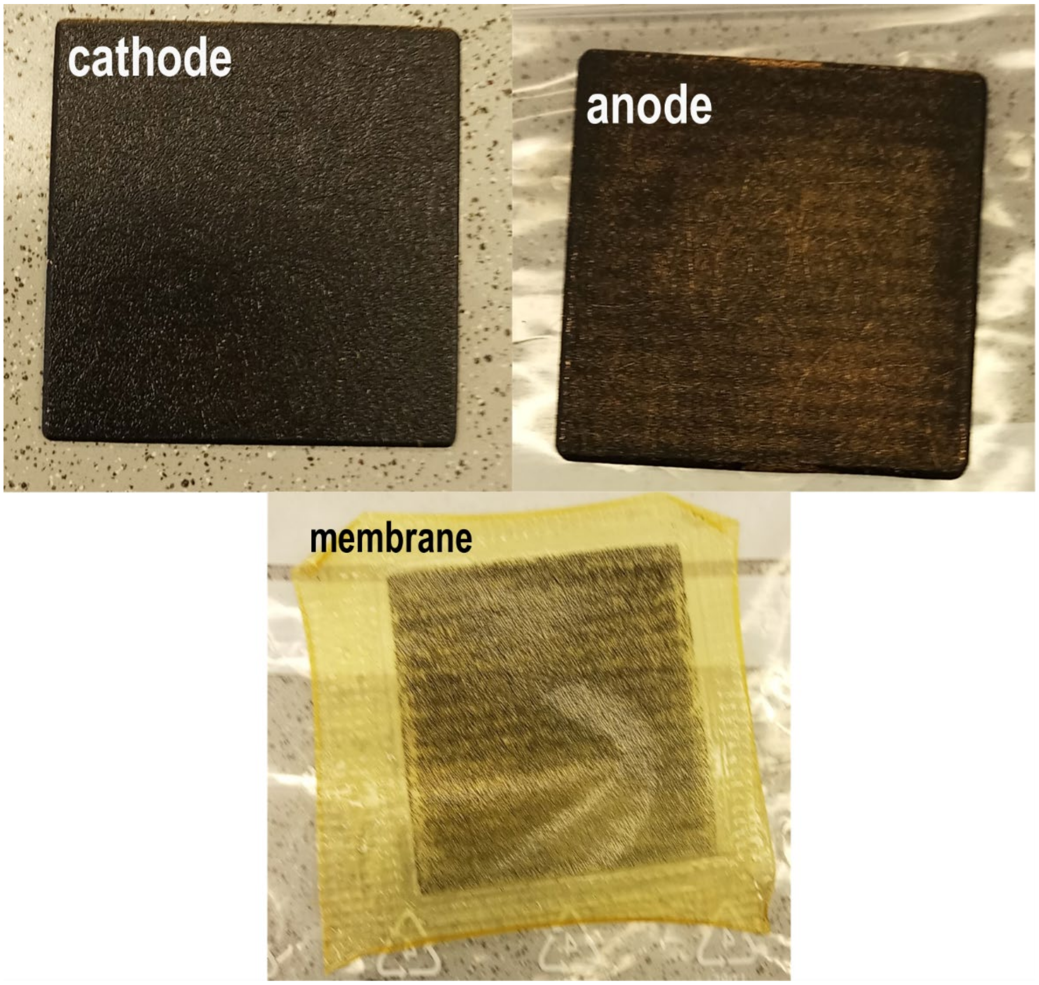

Figure S11: Photographs of anode and cathode catalyst coated substrates and membrane after stability test.

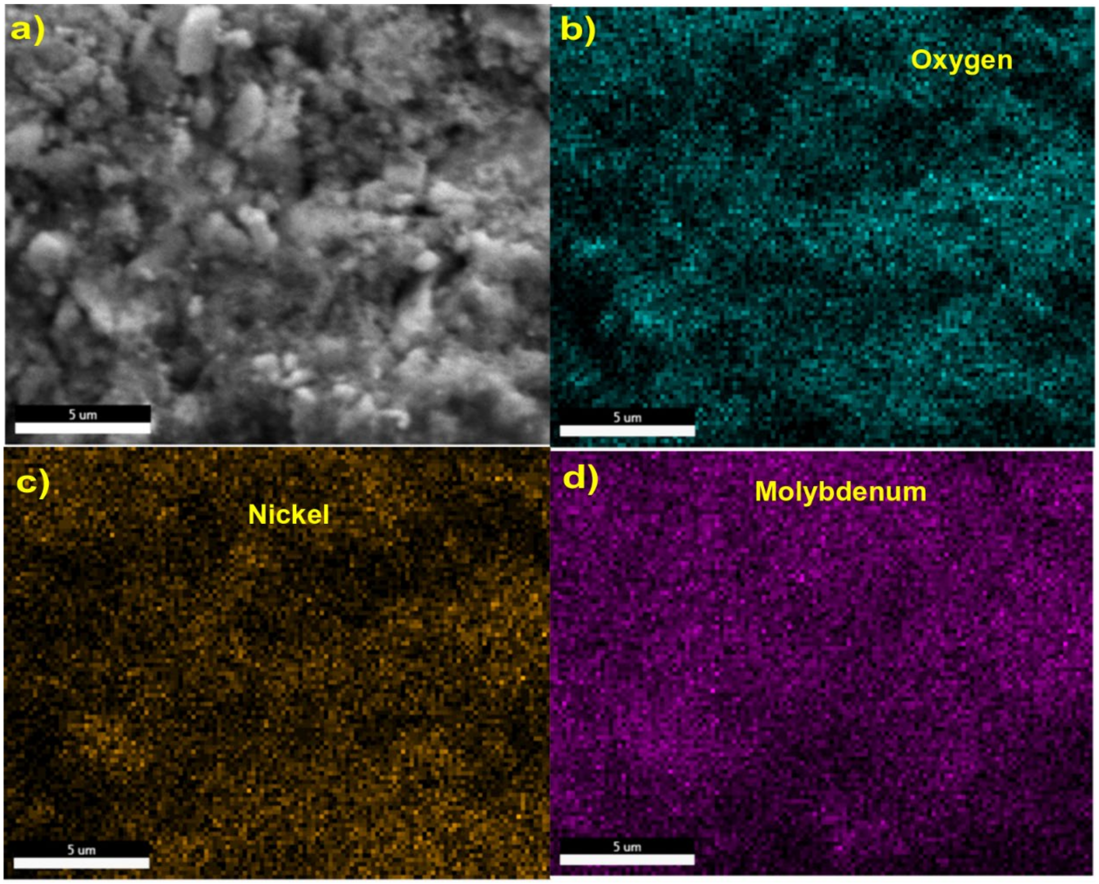

Figure S12: EDX mapping of Ni-MoO_2 nanosheets cathode catalytic layer after stability test.

Table S2: Parameters extracted from impedance data fitting for the durability of Ni-MoO_2 / $Ni_{0.6}Co_{0.2}Fe_{0.2}$ electrolysis cells at the beginning of the stability test (BOT) and the end of the stability test (EOT) after 65 hours.

Impedance fitting	BOT	EOT
R_Ω	0.3025	0.3382
Q1	158.9	209.475
A1	0.791	0.644
R_{ct}	0.2376	0.3615
Q2	25.3	57.6
A2	1	1
R1	0.1143	0.15

References

(1) Ayers, K.; Capuano, C.; Atanassov, P.; Mukerjee, S.; Hickner, M. *High Performance Platinum Group Metal Free Membrane Electrode Assemblies through Control of Interfacial Processes*; Golden, CO (United States), 2017. https://doi.org/10.2172/1410560.

(2) Kraglund, M. R.; Carmo, M.; Schiller, G.; Ansar, S. A.; Aili, D.; Christensen, E.; Jensen, J. O. Ion-Solvating Membranes as a New Approach towards High Rate Alkaline Electrolyzers. *Energy Environ. Sci.* **2019**, *12* (11), 3313–3318. https://doi.org/10.1039/c9ee00832b.
